# Genome-wide identification, gene cloning, subcellular location and expression analysis of the *OPR* gene family under salt stress in sweetpotato

**DOI:** 10.1186/s12870-024-05887-8

**Published:** 2024-12-06

**Authors:** Wenxing Li, Yongping Li, Yuan Xu, Sunjeet Kumar, Yi Liu, Guopeng Zhu

**Affiliations:** 1 Sanya Nanfan Research Institute, Hainan University, Sanya, 572025 China; 2 Key Laboratory for Quality Regulation of Tropical Horticultural Crops of Hainan Province, School of Tropical Agriculture and Forestry, Hainan University, Haikou, 570228 China

**Keywords:** Sweetpotato, *OPR* gene family, Gene cloning, Subcellular localization, Gene expression

## Abstract

**Background:**

The 12-oxo-phytodienoic acid reductase (OPR) enzyme is crucial for the synthesis of jasmonates (JAs), and is involved in the plant stress response. However, the *OPR* gene family in sweetpotato, an important horticultural crop, remains unidentified.

**Results:**

In this study, we employed bioinformatics techniques to identify nine *IbOPR* genes. Phylogenetic analysis revealed that these genes could be divided into Group I and Group II. Synteny analysis indicated that IbOPR evolution was driven by tandem duplication, whole-genome duplication (WGD), and segmental duplication events. The promoter sequences of *IbOPRs* were found to be associated with stress and hormonal responses. Additionally, we successfully cloned four *IbOPRs* from "Haida HD7791" and "Haida HD7798" using homologous cloning technology. These sequences were 1203 bp, 1200 bp, 1134 bp, and 1137 bp in length and encoded 400, 399, 377, and 378 amino acids, respectively. The protein sequence similarity between the salt-tolerant variety "Haida HD7791" and the salt-sensitive variety "Haida HD7798" was determined to be 96.75% for IbOPR2, 99.75% for IbOPR3, 92.06% for IbOPR6, and 98.68% for IbOPR7. Phylogenetic analysis categorized IbOPR2 and IbOPR3 proteins into Group II, while IbOPR6 and IbOPR7 proteins belonged to Group I. Subcellular localization experiments showed IbOPR2 protein present in the peroxisome, while IbOPR3, IbOPR6, and IbOPR7 proteins were found in the cytoplasm and nucleus. Salt stress induction experiments demonstrated that *IbOPR2*, *IbOPR3*, and *IbOPR7* were significantly upregulated only in 'Haida HD7791' after 6 h. In contrast, *IbOPR6* was induced in 'Haida HD7798' at 6 h but inhibited in 'Haida HD7791' at later time points (12, 24, 48, and 72 h), highlighting functional differences in salt stress responses.

**Conclusions:**

Our findings suggest that *IbOPR2* may play a crucial role in sweetpotato's response to salt stress by participating in JAs synthesis. These results provide a foundation for future functional analyses of *OPR* genes in sweetpotato.

**Supplementary Information:**

The online version contains supplementary material available at 10.1186/s12870-024-05887-8.

## Introduction

JAs are a class of plant hormones that play essential roles in regulating defense and development processes [1]. JAs are essential signaling compounds mediating the plant response to salt stress [2]. Studies have demonstrated that the exogenous application of JA significantly reduces the Na^+^ ion content in salinity-tolerant rice and wheat [3–5]. In addition, Methyl jasmonate (MeJA), a JA derivative, has been found to counteract the adverse effects of salt stress on plant growth, chlorophyll content, leaf photosynthetic rate, leaf transpiration rate, and proline content [6]. Furthermore, MeJA has been reported to alleviate the harmful effects of salinity stress on German chamomile by increasing the activity of antioxidant enzymes [7]. Together, these findings demonstrate the important role of JAs in mediating plant adaptation to salt stress and suggest the potential usefulness of JAs in improving crop productivity under adverse conditions.

The biosynthesis of JA is initiated by the release of α-linolenic acid (18:3) from galactolipids in the chloroplast membranes. A 13-LOX enzyme then oxygenates this acid to form *cis*-( +)-12-oxo-phytodienoic acid (OPDA) within the chloroplast through the activity of allene oxide synthase (AOS) and allene oxide cyclase (AOC). A similar conversion process occurs with hexadecatrienoic acid (16:3), which leads to dinorOPDA (dnOPDA), another potential precursor of JA. The peroxisomal ABC transporter is responsible for transporting OPDA and dnOPDA from the chloroplast to the peroxisomes after their formation. In peroxisomes, the cyclopentenone rings of OPDA and dnOPDA are further reduced by OPR, which converts them to 8-(3-oxo-2(pent-2-enyl) cyclopentenyl) octanoic acid (OPC-8) and OPC-6, respectively. OPC-8 is then converted into OPC-6 and OPC-4 derivatives through the fatty acid β-oxidation pathway, eventually producing JA in the peroxisomes. JA is then released into the cytosol [8]. This biosynthetic pathway provides a general overview of the mechanisms involved in JA synthesis.

The OPR belongs to the old yellow enzyme family of flavoenzymes and forms multiple subfamilies in angiosperm plants [9]. A typical OPR protein has only one Oxidore-FMN domain. To date, 3, 13, 48, 5, 7, 10, and 8 *OPR* genes have been identified in *Arabidopsis thaliana* [10], rice [11], wheat [12], watermelon [13], pepper [14], cotton [15], and *Zea mays* [16], respectively. Based on sequence similarity, the OPR family of higher plants is classified into five subgroups (I-V): dicotyledons have only subgroups I and II, while monocotyledons have all five [9]. In previous studies, based on substrate specificity, the OPRs in *Arabidopsis thaliana* were divided into two groups: Group I (OPRI) and Group II (OPRII) [17]. The AtOPR1 and AtOPR2 enzymes preferentially catalyze (9R,13R)-12-oxophytodienoic acid (9R,13R-OPDA), which is not involved in the JA biosynthetic process. In contrast, AtOPR3 is capable of converting 9S,13S-OPDA to oxo-2(2’(Z)-pentenyl)-cyclopentane-1-octanoic acid (OPC-8:0), which is a precursor of JA biosynthesis [10, 18]. AtOPR3 is thought to be involved in JA synthesis, while AtOPR1 and AtOPR2 are not.

*OPR* genes play an important role in the abiotic stress response. For instance, in wheat, overexpression of *TaOPR1* has been found to enhance plant tolerance to salinity in an ABA-dependent manner [19]. Similarly, overexpression of *OsOPR7* in tobacco has been shown to alleviate salinity-induced mitochondrial oxidative damage [20]. Furthermore, transgenic wheat plants with increased *AtOPR3* expression have delayed germination, growth, blooming, and senescence. Short-term cold tolerance is also improved [21]. These findings suggest that *OPR* genes may have potential applications in crop improvement under adverse environmental conditions.

Sweetpotato (*Ipomoea batatas* (L.) Lam) is a hexaploid (2n = 6x = 90) species rich in calories, proteins, vitamins, and minerals. It is the seventh most important crop in the world, and the fourth most significant crop cultivated in China [22]. However, salt stress is a significant environmental stress limiting plant growth and productivity [23]. To adapt to salt stress, sweetpotato enhances salt tolerance by increasing the contents of betaine, proline, abscisic acide (ABA), and activating the clearance of reactive oxygen species, promoting inositol biosynthesis, photosynthesis, and ion balance [24–28]. Recent studies have also shown that JA plays important roles in sweetpotato’s responses to salt stress. Specifically, JA can enhance the salt tolerance of sweetpotato by regulating stomatal closure and maintaining ion homeostasis [29]. In a previous study, the exogenous application of MeJA was found to reduce the damage caused by salt stress in sweetpotato “Haida HD 7791” [30]. This study aimed to identify the *IbOPR* gene family, which plays a critical role in JA synthesis but has not been previously reported in sweetpotato. Through bioinformatics methods, we identified and characterized nine putative *IbOPR* genes using the whole-genome data of the sweetpotato variety “Taizhong 6”. We analyzed their physical and chemical characteristics, gene structure, conserved motifs, chromosome localization, *cis*-acting regulatory elements, and evolutionary relationships. Furthermore, to understand the role of *IbOPRs* in salt stress responses, we cloned four *IbOPR* genes and examined their gene expression levels under 200 mM NaCl treatment using RT‒qPCR. This analysis provided insights into the function of *IbOPRs* in combating salt stress in sweetpotato. These findings contribute to the identification of potential functional genes that could be utilized to enhance salt stress resistance in sweetpotato.

## Results

### Identification and characterization of *IbOPRs*

**Fig. 1:**
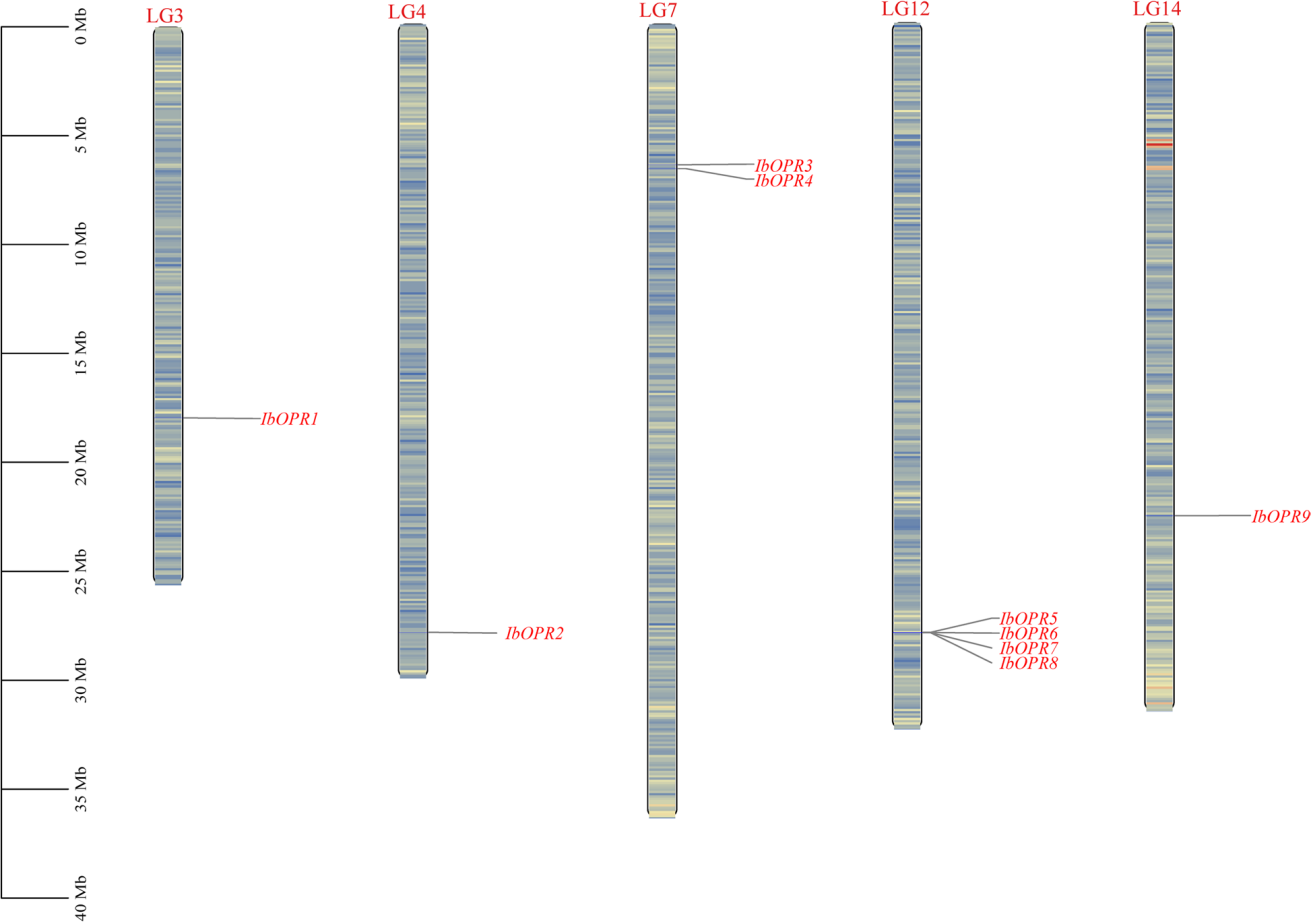
Gene location of the *IbOPRs*. The number in the left represent the length of LG

Initially, the BLAST and HMMER search methods were used to identify putative *OPR* genes in sweetpotato, followed by further confirmation using the NCBI conserved domains database. This approach resulted in the identification of nine *IbOPR* genes in the sweetpotato genome. The chromosomal location of these *IbOPR* genes were determined, showing that the nine identified *IbOPR* genes were distributed across LG3, 4, 7, 12, and 14 (Fig. 1). These nine genes were named *IbOPR1-9* according to their position within the Linkage Groups (LGs).

Our analysis revealed that the length of IbOPR proteins varied slightly, with the shortest protein, IbOPR9, encoding 318 amino acids, and the longest protein, IbOPR4, encoding 442 amino acids. The molecular weights of the proteins ranged from 34.97 kDa to 44.15 kDa. The isoelectric point ranged from 5.43 (IbOPR9) to 8.78 (IbOPR2), with eight proteins being acidic and one protein being basic. The scores indicated that all IbOPR proteins were hydrophilic (GRAVY < 0) (Table S1). These findings provide a theoretical basis for further research into the purification, activity, and function of IbOPR proteins.

Furthermore, the predicted subcellular localization of the IbOPR proteins showed that the IbOPR2, IbOPR3, and IbOPR4 proteins were located in the peroxisome, while the IbOPR1, IbOPR5, IbOPR6, IbOPR7, IbOPR8, and IbOPR9 proteins were found in the cytoplasm (Table S1).

### Phylogenetic analysis of *OPR* proteins

**Fig. 2:**
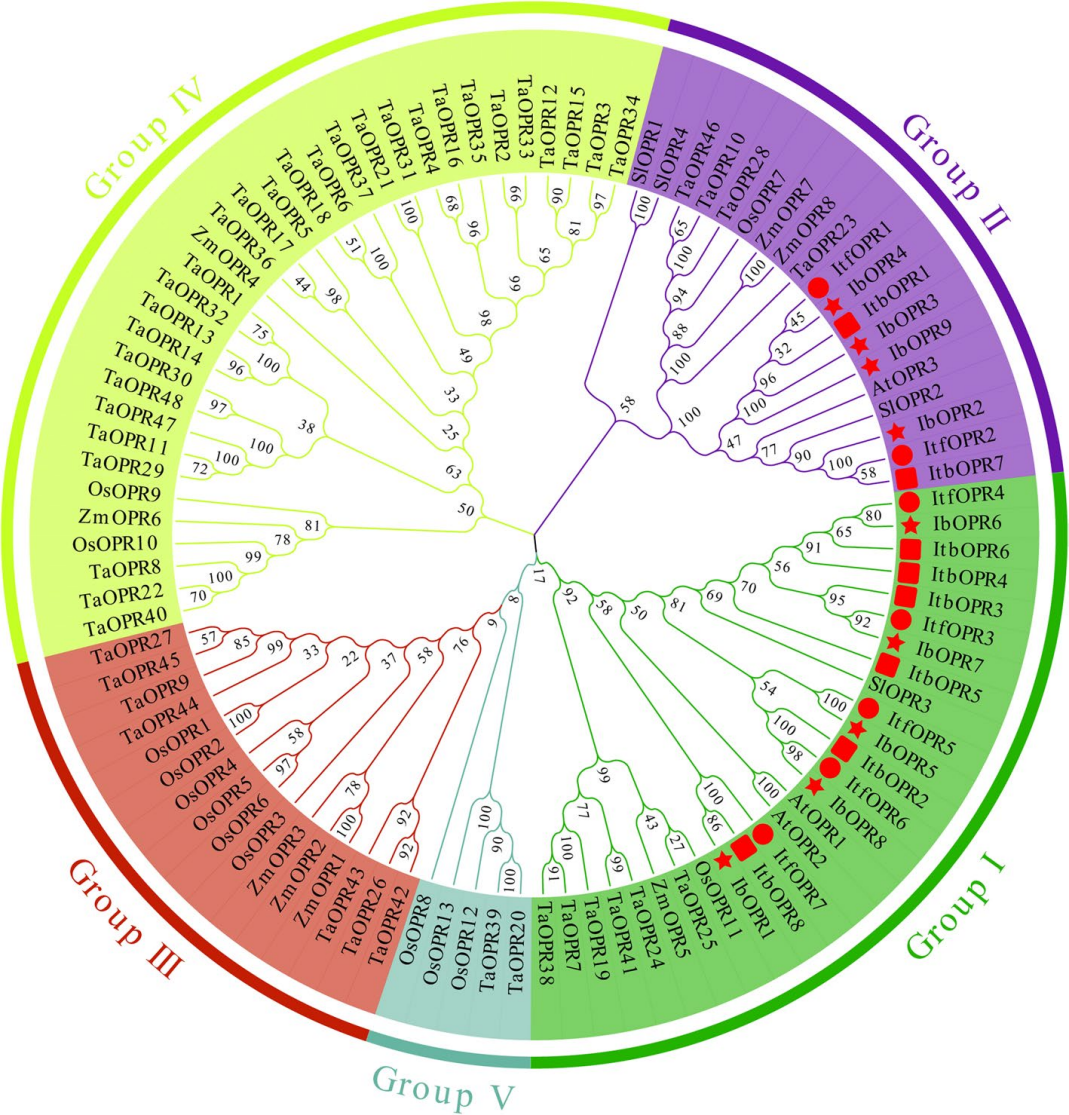
The maximum likelihood (ML) phylogenetic tree of the OPR family. The tree was drawn with the full-length amino acid sequences of OPR protein from *Arabidopsis thaliana* (Linn.) Heynh. (At), *Ipomoea batatas* L. (Ib), *Oryza sativa* L. (Os), *Solanum lycopersicum* L. (Sl), *Triticum aestivum* L. (Ta), and *Zea mays* L. (Zm), *Ipomea triloba* (Itb), *Ipomea trifida* (Itf), using MEGA 7.0, with 1000 replicates. The red rect, circle and star represented the OPR protein from *Ipomea triloba*, *Ipomea trifida*, and *Ipomoea batatas* L, respectively

To elucidate the taxonomic and evolutionary connections within the IbOPR family, we utilized MEGA7.0 to construct a maximum likelihood (ML) phylogenetic tree. The tree consisted of 9 IbOPRs, 8 ItbOPRs, 7 ItfOPRs, 13 OsOPRs, 3 AtOPRs, 8 ZmOPRs, 4 SlOPRs, and 48 TaOPRs, as depicted in Fig. 2. The phylogenetic tree classified the 100 OPRs into five different groups. For reference, the names and protein sequences of OPRs from different species are provided in Table S2.

The results showed that the 100 OPR proteins were classified into five groups: Group I contained 27 OPR proteins, Group II contained 19, Group III contained 16, Group IV had 33, and Group V had 5. Group IV was the largest, and Group V was the smallest. Monocot species had OPR members in all five groups, while dicot species were only clustered in Groups I and II, consistent with a previous study [9]. Within the IbOPR proteins, a distinct grouping was observed. IbOPR1, IbOPR5, IbOPR6, IbOPR7, and IbOPR8 were classified under Group I and were found to cluster together with AtOPR1 and AtOPR2 in the same clade. On the other hand, IbOPR2, IbOPR3, IbOPR4, and IbOPR9 belonged to Group II and exhibited a close relationship with AtOPR3.

### Gene structure and conserved motif analysis of *IbOPR*

**Fig. 3:**
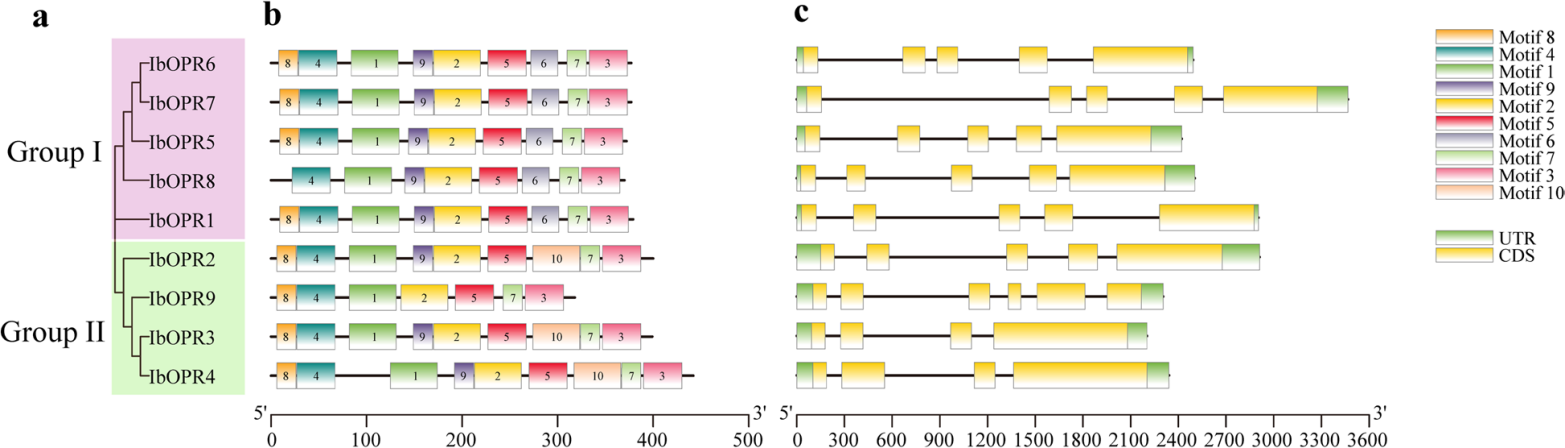
Phylogenetic, gene structural, and conserved motif analysis of IbOPRs. **a** The phylogenetic tree of IbOPR proteins. The MJ phylogenetic tree was constructed by using MEGA 7.0 with 1000 replicates. **b**The distribution of conserved motifs of IbOPR proteins by the MEME. Different color boxes represent ten conserved motifs. **c** Exon–intron structures of *IbOPR* genes. Yellow boxes mean exons, green boxes mean UTRs and black lines mean introns

Typically, genes clustered together in a subgroup have a similar structure. Our study found that the *OPRs* in Group I contained four introns, while Group II consisted of *OPRs* with three to five introns (Fig. 3a, and Fig. 3c). Different subgroups differed in the quantity and length of introns and exons. The *OPR* gene family in sweetpotato underwent loss and gain of introns and exons, particularly in Group II. Our results indicate that the exon–intron ratio of the *IbOPR* genes has mainly remained consistent throughout their evolutionary history.

To identify the conserved motifs of OPR proteins in sweetpotato, we used MEME online software combined with TBtools. Motifs 1–10 were identified as potential motifs (Fig. 3b, and Table S3). These motifs ranged in size from 21 to 50 amino acids, with 7 to 9 motifs found in IbOPR family members. The majority of the orthologous proteins in the same group shared comparable motif members. In Group I, for example, all IbOPRs possessed Motifs 1–9 except IbOPR8, which lacked Motif 8. Except for IbOPR9, which lacked Motifs 9 and 10, all IbOPRs in Group II possessed Motifs 1–5 and 7–10. Notably, each group has its specific motif, for example, Motif 6 in Group I and Motif 10 in Group II. Overall, our findings confirm the classification of IbOPR proteins and show that motifs in these proteins are conserved.

### Collinear analysis, gene duplication, and Ka/Ks analysis

**Fig. 4:**
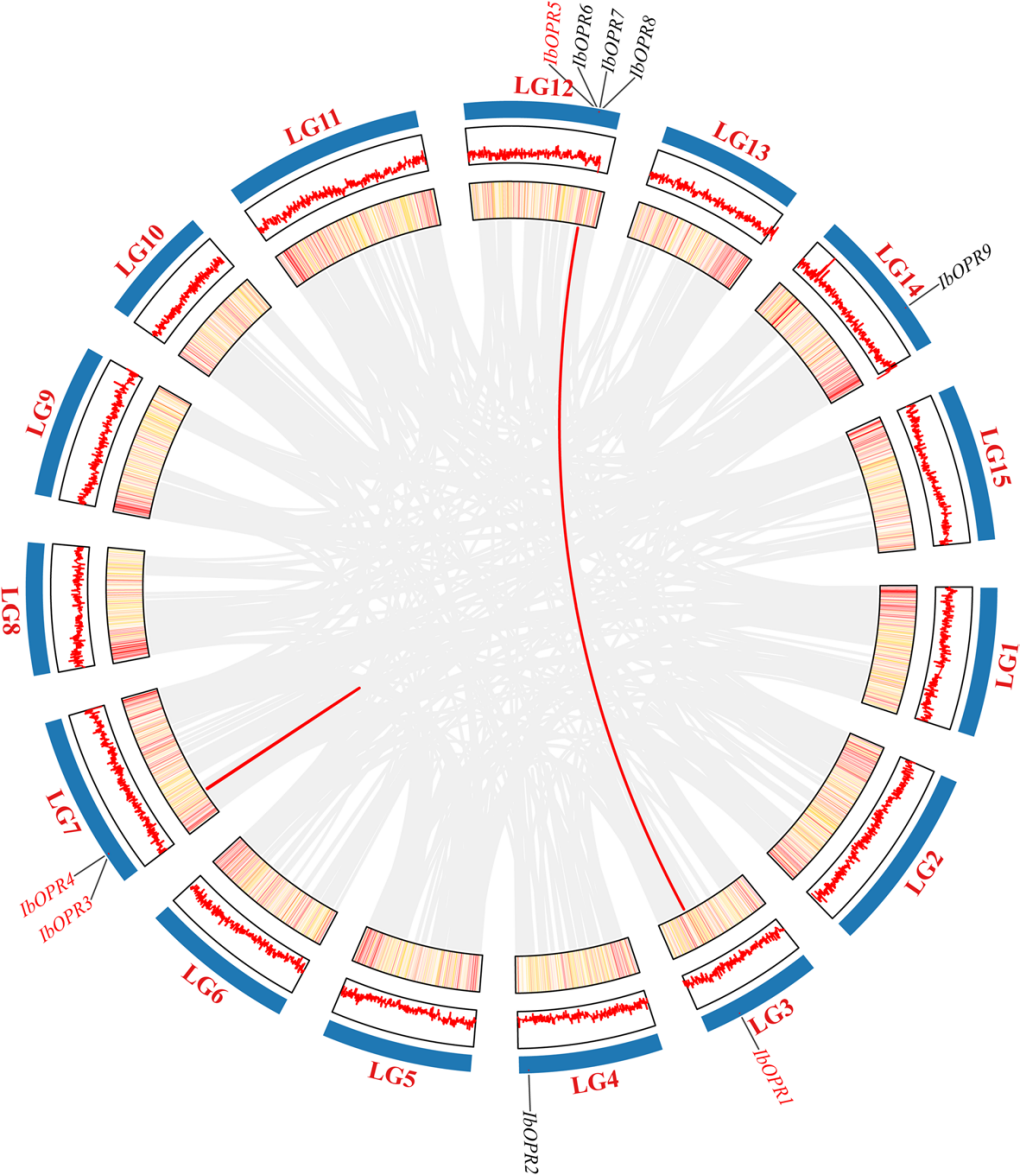
The collinearity analysis of the *IbOPR* genes. The red lines represent the WGD, or segmental duplication gene pair between chromosomes, and the gray lines represent the synteny blocks of the genes in sweetpotato genome. The line and heatmap in the outer circle represented gene density on the chromosome

Gene replication is one of the main drivers of genome evolution, and promoting gene expansion. The main modes of gene replication include whole-genome duplication, tandem duplication, segment duplication, and reverse transcriptional duplication which lead to the increase and diversification of the genome. Four *IbOPR* genes (*IbOPR5*/*IbOPR6*, *IbOPR6*/*IbOPR7*, and *IbOPR7*/*IbOPR8*) were clustered into three tandem duplication events on LG12. Additionally, 2 WGD (whole genome duplication) or segmental duplication events involving 4 *IbOPR* genes (*IbOPR3*/*IbOPR4* and *IbOPR1*/*IbOPR5*) were also identified with MCScanX methods (Fig. 4). These events suggest that tandem duplication, WGD, or segmental duplication played an important role in the expansion of the *IbOPR* gene family. To understand the mechanism of gene divergence and evolutionary pressure, we also determined the Ka/Ks ratios (Table S4). A Ka/Ks ratio greater than 1 indicates positive selection, making the gene susceptible to nonsynonymous mutations. If the ratio equals 1, it means neutral selection, while a ratio less than 1 suggests purifying selection and preferential occurrence of synonymous mutations. All Ka/Ks values were less than 1, suggesting that the *IbOPR* genes underwent purifying solid selection during evolution.

### *Cis*-element analysis in the promoter of *IbOPR* genes in sweetpotato

**Fig. 5:**
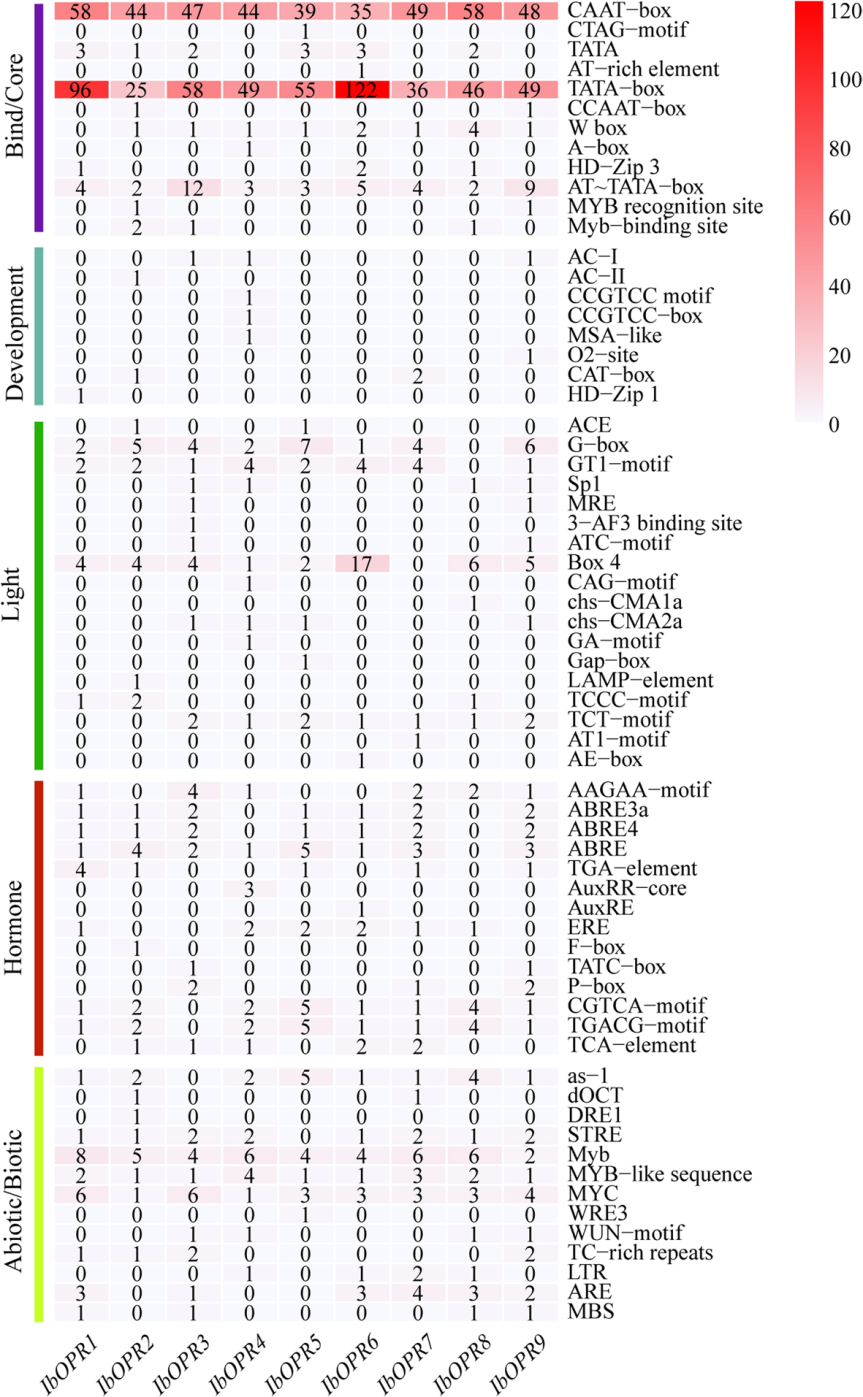
*Cis*-elements analysis of *IbOPRs* in sweetpotato. The *cis*-elements were divided into five broad categories. The degree of red colors represented the number of *cis*-elements upstream of the *IbOPRs*

*Cis*-elements are important elements located upstream of the gene start codon that plays a crucial role in gene function involved in plant development and stress responses. We extracted 2000 bp upstream sequences of *IbOPR* genes in sweetpotato and performed *cis*-element analysis. The elements were grouped into five categories: core/binding, development, light, hormone, and abiotic/biotic factors (Fig. 5 and Table S5). All nine *IbOPR* genes had many core promoter elements and binding sites, such as the TATA box, CCAAT box, AT-TATA box, W box, and MYB recognition site. Light-responsive elements, such as Box4, GT1-motif, G-box, and TCT-motif, were found in most *IbOPR* genes. Moreover, some development-related elements were found in *IbOPR* genes. For example, MSA-like, which is related to cell cycle regulation, was found only in *IbOPR3*; the CAT-box, which is associated with meristem formation and cell division, was found in *IbOPR1* and *IbOPR7*; the O2-site, which is associated with zein metabolism, was found in *IbOPR6* and *IbOPR9*. In addition, hormone-responsive elements were abundant in *IbOPR* gene*s*, including the ABA-responsive elements ABRE and AAGAA-motif; MeJA-responsive elements CGTCA-motif and TGACG-motif; SA-responsive element TCA-element; GA-responsive elements F-box, P-box, and TATC-box; ETH-responsive element ERE; and IAA-responsive elements AuxRE, AuxRR-core, and TGA-element. In addition, some abiotic-responsive elements, such as drought and salt-responsive elements, including MBS, Myb, MYC, anaerobic induction element ARE, and low temperature-responsive element LTR, were found in most *IbOPR* genes. Overall, these results indicate that *IbOPR* genes are involved in regulating plant growth, development, and stress adaptation in sweetpotato.

### Cloning and sequence analysis of *IbOPR*

**Fig. 6:**
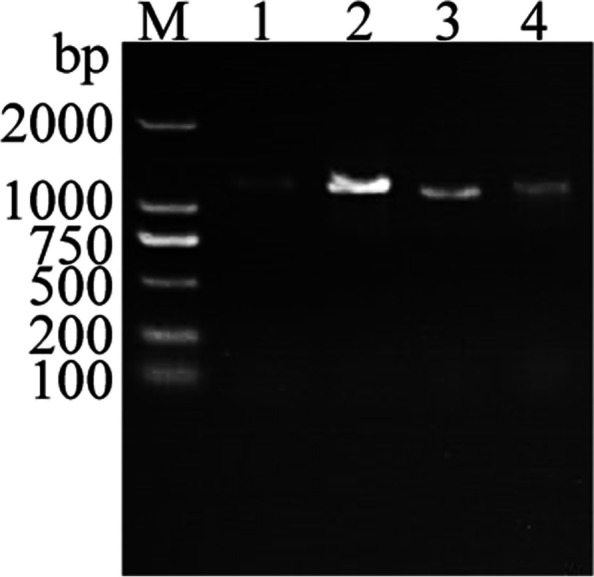
PCR amplification of *IbOPR2*, *IbOPR2*, *IbOPR6*, and *IbOPR7* genes. The excess gel is cropped in the image; M represents DNA marker, 1–4 represents *IbOPR2*, *IbOPR3*, *IbOPR6*, and *IbOPR7*

**Fig. 7:**
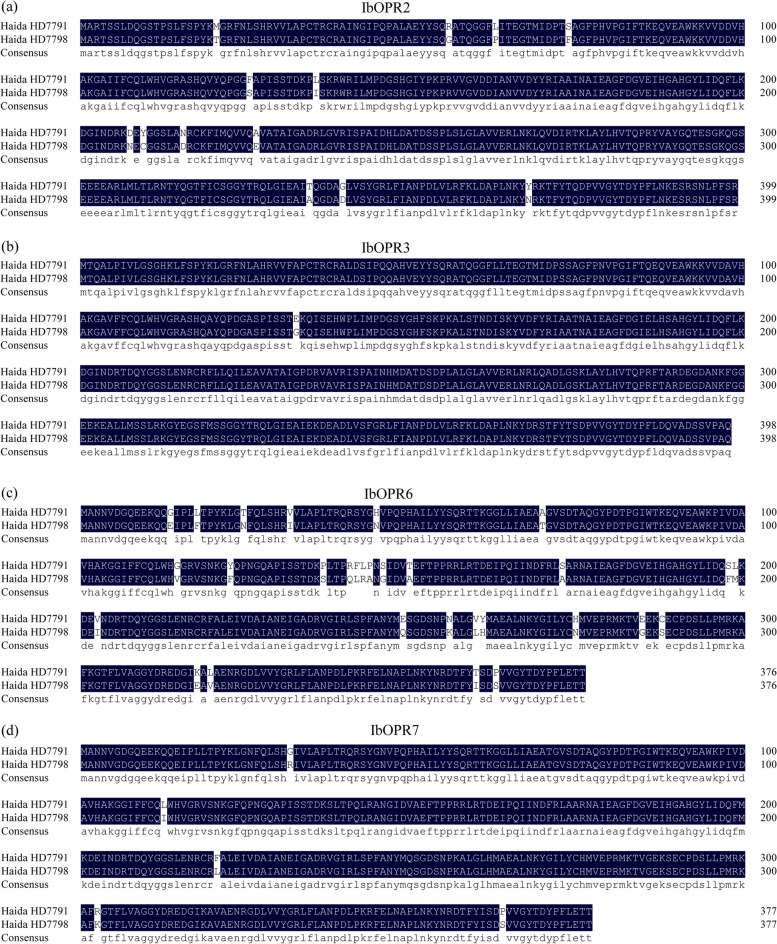
Protein sequence comparison of IbOPR2, IbOPR3, IbOPR6, IbOPR7 between “Haida HD7791” and “Haida HD7798”

According to the reference coding sequence (CDS) of the nine *IbOPR* genes (Table S6)*,* using “Haida HD7791” and “Haida HD7798” cDNA as templates, *IbOPR* primers were used for PCR amplification (Table S7). The electrophoresis bands were consistent with the expected sizes of the target fragments (Fig. 6, Fig. S1). *IbOPR2*, *IbOPR3*, *IbOPR6*, and *IbOPR7* were eventually cloned. The coding region sequences of *IbOPR2, IbOPR3, IbOPR6,* and *IbOPR7* from “Haida HD7791” and “Haida HD7798” were obtained by sequencing and were 1203, 1200, 1134, and 1137 bp, encoding 400, 399, 377, and 378 amino acids, respectively (Table S6). The CDS similarity between “Haida HD7791” and “Haida HD7798” for *IbOPR2*, *IbOPR3*, *IbOPR6*, and *IbOPR7* were found to be 98.25%, 99.92%, 95.06%, and 98.50%, respectively (Fig. S2). The protein sequence similarity values for IbOPR2, IbOPR3, IbOPR6, and IbOPR7 between “Haida HD7791” and “Haida HD7798” were 96.75%, 99.75%, 92.06%, and 98.68%, respectively (Fig. 7).

### Subcellular localization of the IbOPR2 and IbOPR3 proteins

**Fig. 8:**
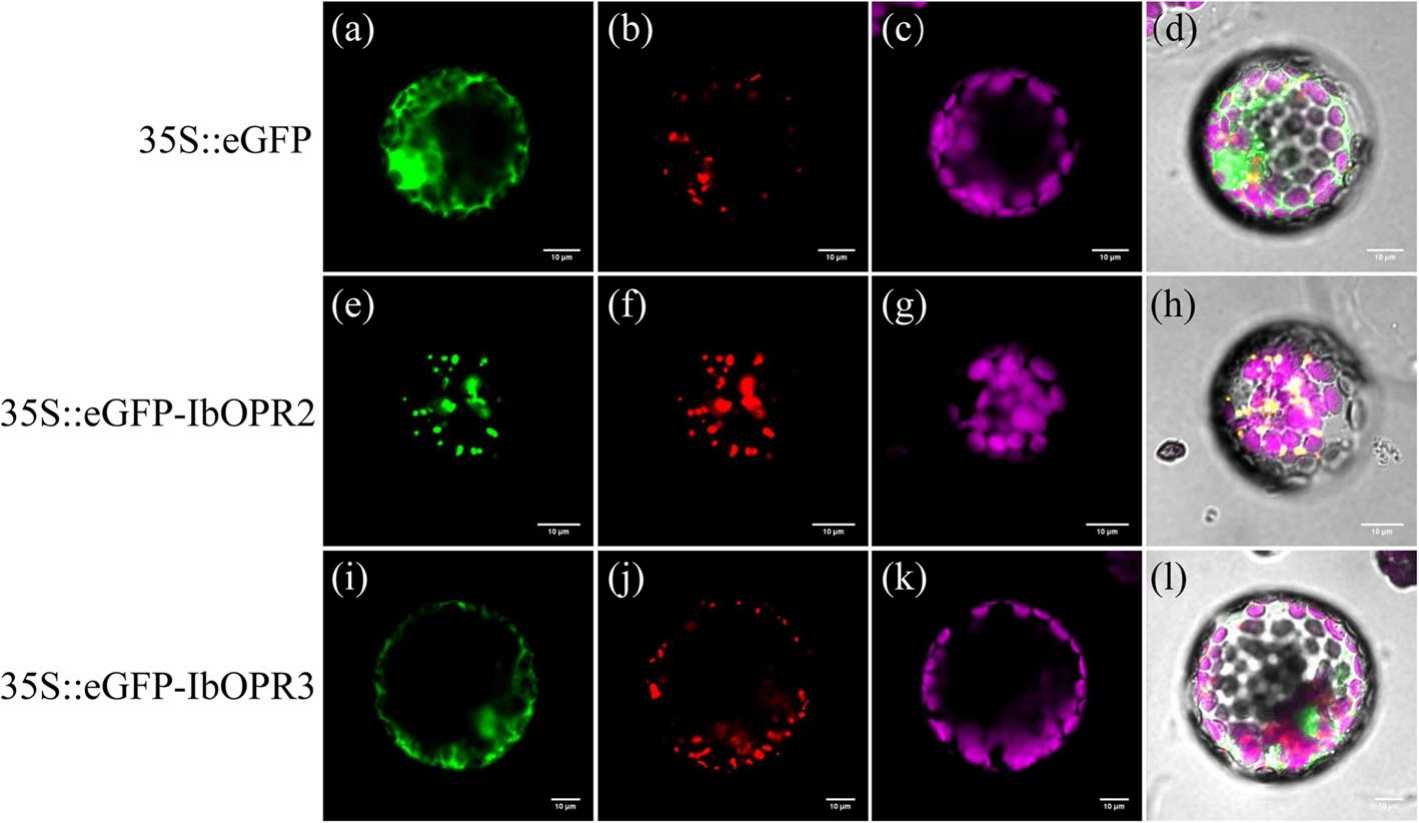
Subcellular localization of sweetpotato OPR2 and OPR3. IbOPR2 and IbOPR3 fusions to eGFP were coexpressed with mKATE-SKL in tobacco protoplast cells via transient expression. eGFP empty vector (**a**-**d**), eGFP-IbOPR2 (**e**–**h**), and eGFP-IbOPR3 (**i**-**l**) colocalize with the peroxisomal marker mKATE-SKL. Images **d**, **h**, and **l** are merged images of the targeted protein fluorescence channel (**a**, **e**, and **i**), mKATE-SKL marker fluorescence channel (**b**, **f**, and **j**), and chloroplast fluorescence channel (**c**, **g**, and **k**). eGFP fusions are in green; mKATE -SKL is in red, chloroplast fluorescence is in magenta. Scale bar = 10 μm

Previous studies have shown that JA is synthesized in peroxisomes, and our subcellular localization prediction indicated that IbOPR2 and IbOPR3 were located in peroxisomes. To further verify the subcellular localization of IbOPR2 and IbOPR3, the enhanced green fluorescent protein (eGFP) was fused to the N-terminus of IbOPR2 and IbOPR3, respectively, and the subcellular localization of the fusion proteins was analyzed by confocal laser scanning microscopy after transient expression in tobacco protoplast cells. Characteristic cytoplasm and nucleus staining were observed when eGFP was expressed alone (Fig. 8a-d). Likewise, the eGFP-IbOPR3 fusion protein showed diffuse cytoplasm and nucleus staining (Fig. 8i-l), indicating that IbOPR3 is localized in the cytoplasm and nucleus instead of peroxisomes. However, cells expressing the eGFP-IbOPR2 fusion exhibited punctuate staining in the cytoplasm (Fig. 8e-h), indicating the association of IbOPR2 with organellar structures. The fluorescence of eGFP-IbOPR2 was found to co-localize with that of the red fluorescent protein mKATE fused to the peroxisomal targeting signal SKL (Ser-Lys-Leu), which is present in hydroxypyruvate reductase, an endogenous enzyme of peroxisomes[31, 32]. The co-localization of IbOPR2 with a bona fide peroxisomal targeting signal indicates that IbOPR2 is also localized in peroxisomes.

### Subcellular localization of the IbOPR6 and IbOPR7 proteins

**Fig. 9:**
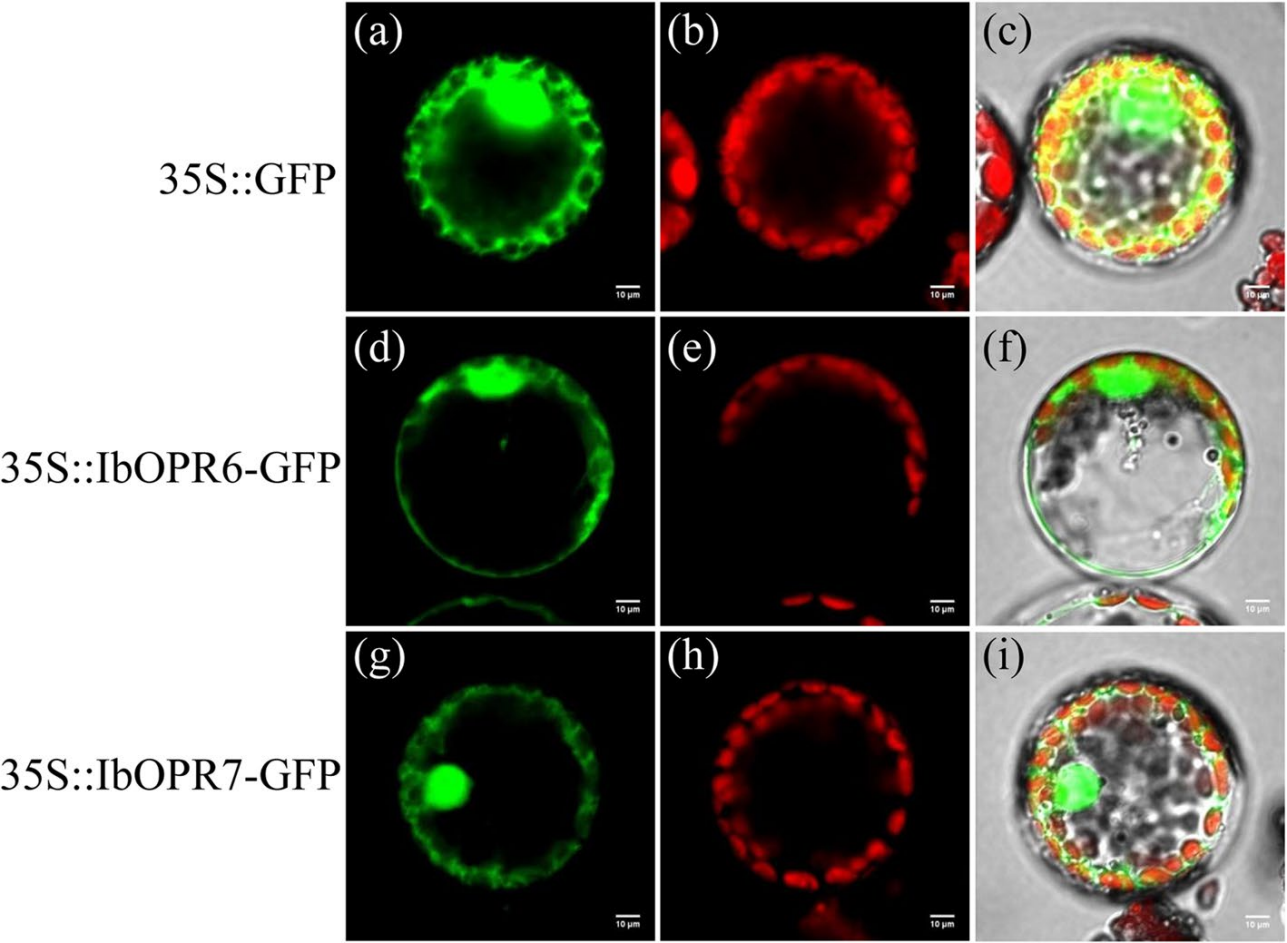
Subcellular localization of sweetpotato OPR6 and OPR7. IbOPR2 and IbOPR3 fusions to eGFP were expressed in tobacco protoplast cells via transient expression. eGFP empty vector (**a**-**c**), IbOPR6-GFP (**d**-**f**), and IbOPR7-GFP (**g**-**i**). Images **c**, **f**, and **i** are merged images of the targeted protein fluorescence channel (**a**, **d**, and **g**) and chloroplast fluorescence channel (**b**, **e**, and **h**). GFP fusions are in green; chloroplast fluorescence is in red. Scale bar = 10 μm

Our subcellular localization prediction found that IbOPR6 and IbOPR7 were located in the cytoplasm. To further validate the result, the green fluorescent protein (GFP) was fused to the C-terminus of IbOPR6 and IbOPR7, respectively, and the subcellular localization of the fusion proteins was analyzed by confocal laser scanning microscopy after transient expression in tobacco protoplast cells. When GFP was expressed alone, characteristic staining patterns were observed in the cytosol and nucleus (Fig. 9a-c). Interestingly, the IbOPR6-GFP and IbOPR7-GFP fusion proteins exhibited similar staining patterns, showing diffuse distribution in the cytoplasm and nucleus (Fig. 9d-f and Fig. 9g-i). These results provide further evidence that IbOPR6 and IbOPR7 are indeed localized in the cytoplasm and nucleus.

### Expression analysis of *IbOPR* genes in response to salt stress

**Fig. 10:**
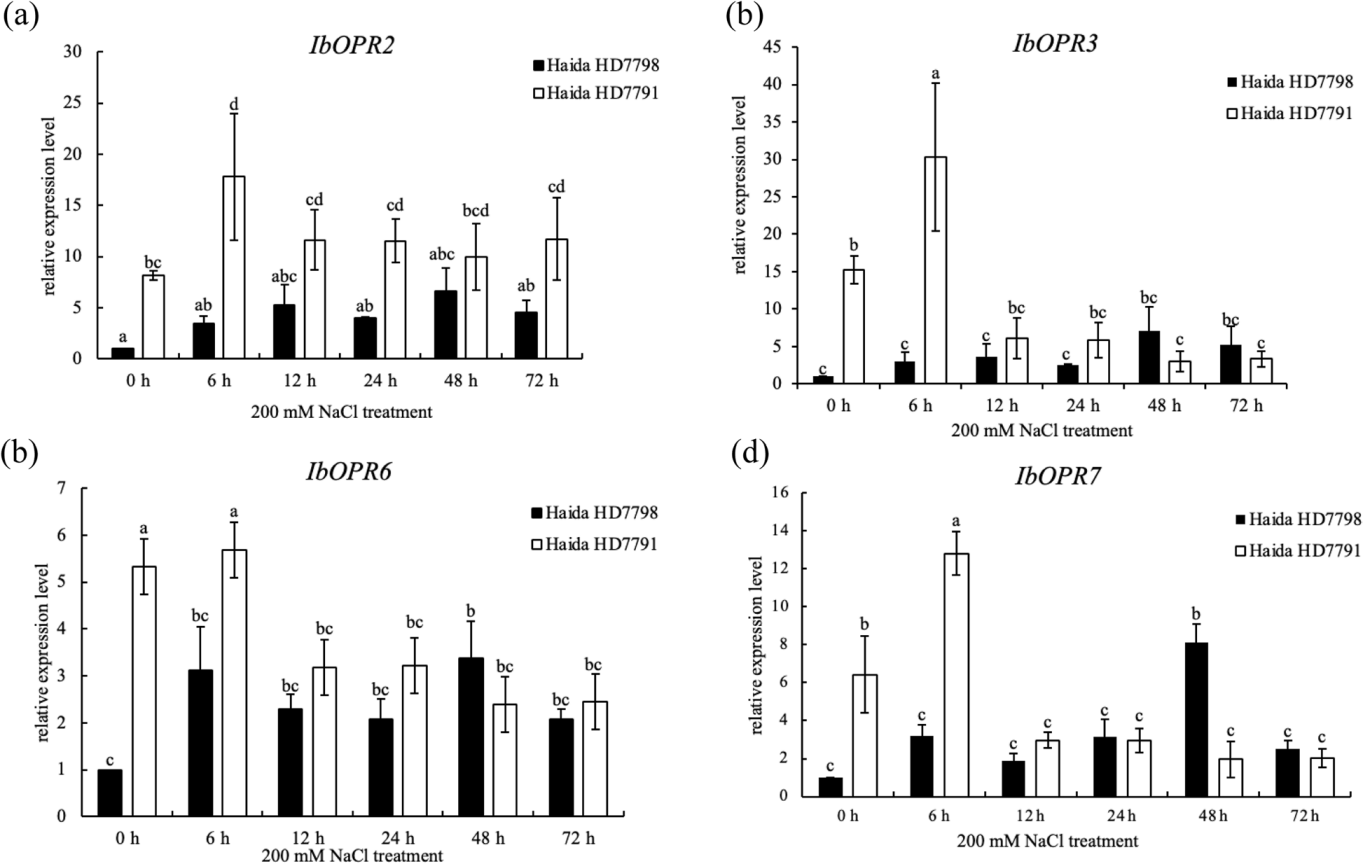
The expression of *IbOPR* genes under 200 mM NaCl. The vertical bar in the bar graph is the standard error. According to the Duncan test, bars with different lowercase letters within each panel are significant differences at *p* < 0.05

To examine the biological function of four *IbOPR* genes in response to salt stress, we analyzed the expression levels of these genes in the salt-tolerant variety “Haida HD7791” and the salt-sensitive variety “Haida HD7798” under 200 mM NaCl treatment by qRT‒PCR (Fig. 10 and Table S8). *IbOPR2*, *IbOPR3*, and *IbOPR7* were not significantly induced by salt stress in “Haida HD7798” but were significantly influenced by 2-, 2- and twofold in “Haida HD7791” at 6 h (*p* < 0.05), respectively. Additionally, the expression of these three genes was significantly higher in “Haida HD7791” than in “Haida HD7798” at 0 h and 6 h (*p* < 0.05). On the other hand, *IbOPR6* was significantly induced by salt stress in “Haida HD7798” at 48 h but was significantly repressed in “Haida HD7791” at 12 h, 24 h, 48 h, and 72 h (*p* < 0.05).

## Discussion

Recent studies have shown that *OPR* genes are crucial in plant growth, development, and stress response [33, 34]. In this study, we identified nine *IbOPR* genes in the sweetpotato “Taizhong6” reference genome using bioinformatics methods. Previous studies showed that the OPR family of monocotyledons is classified into five subgroups, and dicotyledons have only subgroups I and II [9]. Our phylogenetic result is consistent with it. Phylogenetic analysis showed that these nine *OPR* genes were clustered into Groups I and II, indicating that sweetpotato and *Arabidopsis* have a relatively conserved evolutionary history [10, 35, 36]. Furthermore, gene structure research showed that most *IbOPR* genes in a group had a similar exon/intron structure, suggesting that evolution may have affected gene function and structure [37]. Intron loss and gain can contribute to generating structural diversity and complexity [38]. *IbOPR* genes in Group I had four introns, whereas Group II had 3 to 5 introns. In contrast, 33 of the 48 *TaOPR* genes had fewer than three introns, and *ZmOPR1*, *ZmOPR2*, and *ZmOPR3* had only one intron [12, 16].

The results indicate that intron loss events occurred independently in various plant lineages, including sweetpotato, wheat, and maize. The gene structure analysis corroborates these findings, revealing that most *IbOPR* genes within the same group share common protein motifs. It was observed that all IbOPR proteins possess Motifs 1–5. In Group I, Motif 6 is present in all members, whereas none of the Group II members exhibit this motif. Conversely, in Group II, all members have Motif 10, while none of the Group I members possess it. The absence of these specific motifs in the respective groups is crucial for maintaining the secondary structure of the protein [39]. The similarity in gene structure and motif composition within subgroups suggests a common ancestral origin and potential functional similarities among these *IbOPR* genes.

The roles of *cis*-acting elements in influencing gene expression have gained emphasis in recent years [40]. This study found that all *IbOPR* gene promoters contained multiple *cis*-acting elements associated with stress and hormonal responses, such as salicylic acid (SA), MeJA, ethylene (ETH), ABA, Indole-3-acetic acid (IAA), and gibberellin (GA). This suggests that the *IbOPR* gene family may play a role in sweetpotato’s response to stress and development. In particular, hormone-induced related elements such as SA, MeJA, ETH, and ABA indicated that *IbOPR*s were related to the stress of sweetpotato. Previous studies have shown that the OPR protein can be regulated by protein kinases or transcription factors. As an example, GhCPK33 was responsible for phosphorylating GhOPR3, resulting in the suppression of JA accumulation. Similarly, TaFBA-2A, an F-box transcription factor, facilitated the degradation of TaOPR2 through the ubiquitin-26S proteasome pathway, leading to the negative regulation of JA biosynthesis [41, 42]. The *IbOPR* promoters had multiple binding sites *cis*-acting elements, such as the W-box, Myb-binding site, and MYB recognition site. WRKY proteins can bind to the W-box in the promoter of their target genes and activate or repress the expression of downstream genes, thereby regulating their stress response [43, 44]. Therefore, we speculated that WRKY, MYB transcription factors, etc., could regulate *IbOPR*.

Among the 9 *IbOPR* genes, 7 *IbOPR* genes clustered into three tandem duplication events and 2 WGD, or segmental duplication events. Similarly, in other plants, such as *TaOPR*, *GhOPR*, and *GbOPR*, tandem duplication events, WGD, or segmental duplication played a major role in the evolution of the *OPR* gene family. Specifically, 37 of 43 *TaOPR* genes, 9 of 10 *GhOPR* genes, and 7 of 9 *GbOPR* genes were clustered into tandem duplication events, whole genome duplication, or segmental duplication [12, 15]. These findings suggest that duplication events have been a key driving force behind the evolution of the *OPR* gene family in monocotyledon and dicotyledon plants.

According to the reference CDS of the nine *IbOPR* genes, we were able to successfully clone *IbOPR2*, *IbOPR3*, *IbOPR6*, and *IbOPR7* from “Haida HD7791” and “Haida HD7798”, but the remaining 5 *IbOPRs* could not be cloned despite trying various methods, such as changing primers, cloning kits, and optimizing the PCR system. Interestingly, the reference CDS of *IbOPR3*, *IbOPR4*, and *IbOPR9* share 223 and 54 of the same bases in the 5’ and 3’ regions, respectively (Fig. S3), so the cloning primer sequences of *IbOPR3*, *IbOPR4*, and *IbOPR9* are identical. In addition, it is worth noting that *IbOPR3*, *IbOPR4*, and *IbOPR9* possess many of the same fragment sequences and base variations. Therefore, our speculation is that *IbOPR3*, *IbOPR4*, and *IbOPR9* are alternative transcripts originating from a single gene. However, during our cloning efforts, we were only successful in isolating *IbOPR3*, indicating its presence, while the genes *IbOPR4* and *IbOPR9* were not detected. We compared CDSs of cloned *IbOPRs* from “Haida HD7791” and “Haida HD7798”. The results revealed nucleotide variations in the four *IbOPR* sequences between the two varieties (Fig. S2). Additionally, there are differences in the encoded protein sequences (Fig. 7). When nucleotide variations occur, they can result in changes in the amino acid sequence during protein synthesis. Consequently, these changes may affect the structure and function of the proteins, resulting in observed differences between the two varieties.

Previous research has revealed that salt stress can stimulate the expression of *CaOPR6* and *GhOPR1* to *GhOPR10*; however, high salinity can suppress the expression of *TaOPR* [13, 15]. To investigate the response of the cloned *IbOPR* genes to salt stress, we analyzed the expression levels of four cloned *IbOPR* genes in the salt-tolerant variety “Haida HD7791” and the salt-sensitive variety “Haida HD7798” under 200 mM NaCl treatment using qRT‒PCR. We determined that salt stress significantly increased the expression of *IbOPR2*, *IbOPR3*, and *IbOPR7* in "Haida HD7791" but not in “Haida HD7798”, and their expression in “Haida HD7791” was significantly higher than that in “Haida HD7798”. In contrast, *IbOPR8* was significantly induced by salt stress in the salt-sensitive variety but significantly suppressed in the salt-tolerant variety. Based on our findings, we predicted that *IbOPR2*, *IbOPR3*, and *IbOPR7* play important roles in the response to salt stresses in sweetpotato. Our previous research showed that MeJA can reduce the damage caused by salt stress in sweetpotato “Haida HD 7791”. In this research, *IbOPR2*, *IbOPR3*, and *IbOPR7* were significantly induced by salt stress in “Haida HD 7791”. The results of the two studies show that sweetpotato can respond to salt stress through the jasmonic acid pathway. Their result is consistent with Zhang et al. [29].

Previous studies have shown that JAs are synthesized in the peroxisomes, and enzymes such as AtOPR3, TaOPR2, and OsOPR7 are involved in JAs synthesis [8, 15, 16, 45]. In our study, subcellular localization prediction showed that Group I members were located in the cytoplasm, while Group II members were primarily located in the peroxisome, except for IbOPR9, which was in the cytoplasm. Subcellular localization experiments confirmed that only IbOPR2 was located in the peroxisome. Moreover, phylogenetic analysis revealed that the IbOPR2, TaOPR2, AtOPR3, and OsOPR7 proteins clustered together in Group II (Fig. 2). Based on this, we predict that the IbOPR2 protein is involved in JAs synthesis in sweetpotato.

In this study, there is no direct evidence to support that IbOPR2 is the key enzyme of JAs synthesis and regulates the salt tolerance of sweetpotato, so the function of *IbOPR2* remains to be further studied. In future research, we will conduct genetic transformation of sweetpotato to verify the function of *IbOPR2*.

## Conclusions

Our investigation successfully identified and characterized nine *IbOPR* genes in sweetpotato. Through phylogenetic analysis, these IbOPR proteins were categorized into two distinct groups, Group I and Group II, demonstrating a consistent distribution of conserved motifs and exon–intron organization within each group. Among the nine *IbOPRs*, four genes (*IbOPR2*, *IbOPR3*, *IbOPR6*, and *IbOPR7*) were isolated from both "Haida HD7791" and "Haida HD7798" sweetpotato varieties. Notably, the gene sequences of these four genes differed between the two varieties. IbOPR2 and IbOPR3 proteins belonged to Group II, while IbOPR6 and IbOPR7 proteins belonged to Group I. The subcellular localization experiment revealed that IbOPR2 protein is specifically located in the peroxisome. This observation led us to speculate that IbOPR2 may be crucial in synthesizing JAs in sweetpotato. To further investigate the potential involvement of these *IbOPRs* in the salt stress response, we conducted qRT‒PCR analysis and found that *IbOPR2*, *IbOPR3*, and *IbOPR7* were induced by salt stress in the salt-tolerant variety. This finding suggests that *IbOPR2*, in particular, plays a vital role in the sweetpotato's response to salt stress by synthesizing JAs.

## Data analysis

All qRT‒PCR data were analyzed with one-way ANOVA, and multiple comparisons were evaluated with Duncan’s test (*p* < 0.05) using the SPSS program (Version 16. Chicago, IL, USA) based on the values of three complete randomized blocks.

## Materials and Methods

### Genome-wide identification of *OPR* genes

The genome sequencing database of *I. batatas* used in this study was based on the genomic data of “Taizhong 6” provided by the Ipomoea Genome Hub (https://sweetpotato.com/). The general feature format files and genome sequences of *Arabidopsis thaliana* were downloaded from TAIR (http://www.arabidopsis.org/). The OPR protein sequence of *Arabidopsis thaliana* served as the reference sequence, which was downloaded from TAIR. It was used as the query sequence to scan by using the Protein Basic Logical Alignment Search Tool (BLASTP, United States National Library of Medicine) with an E-value (≤ 1e^−5^) and identity match (≥ 50%) as thresholds provided by TBtools [46] (https://github.com/CJ-Chen/TBtools). The Hidden Markov Model (HMM) profiles of the Oxidored -FMN (PFAM ID: PF00724) domain were obtained from the Pfam database (https://pfam.xfam.org/) and used as baits to conduct a HMM with an e-value < e-^10^ searching for sequence homologs by using HMMER 3.3.1 [47] (http://hmmer.org/download.html). The candidate *OPR* genes were identified from the integration of BLASTP and HMMER search results and then submitted to the NCBI conserved domains database (https://www.ncbi.nlm.nih.gov/cdd/) [48] to further confirm the presence of Oxidored -FMN domains.

### Physicochemical property analysis of *IbOPR* protein sequences

The basic physicochemical properties of the OPR proteins, including the number of amino acids, molecular weight (MW), protein length, isoelectric point, and grand average of hydropathy (GRAVY), were predicted using the ProtParam tool on ExPASy, an online tool (https://web.expasy.org/protparam/) [49]. Subsequently, the subcellular localization of the OPR proteins was predicted using the online tool (http://www.csbio.sjtu.edu.cn/bioinf/Cell-PLoc-2/) [50].

### Chromosomal localization of *IbOPR* genes

The positions of *IbOPRs* on chromosomes were obtained from the sweetpotato genome annotation information, which was obtained from the *Ipomoea* genome hub (https://sweetpotato.com/). Chromosome mapping was performed and visualized using TBtools [51].

### Conserved motif and gene structure analyses

The MEME online tools (http://meme-suite.org/) (Bailey et al., 2009) were utilized to identify conserved motifs using a discovery motif number of 10 and other default parameters. The gff3 file of the *I. batatas* genome contained information about the gene structure, which was used to identify the exons and introns of OPR. The conserved motif and gene structure can be visualized by TBtools[51].

### Phylogenetic tree construction

According to the Liu et al. [15], We obtained the OPR proteins name of *Arabidopsis thaliana* (Linn.) Heynh. (At), *Oryza sativa* L. (Os), *Solanum lycopersicum* L. (Sl), *Triticum aestivum* L. (Ta), and *Zea mays* L. (Zm) OPR proteins, and search the OPR proteins name in the NCBI to gain the OPR protein sequences. The OPR protein sequences of above species and *Ipomoea batatas* L. (Ib) were aligned by using ClustalW Musle [52], with the default parameters to generate a neighbor-joining tree. The maximum likelihood phylogenetic tree was constructed with all OPR protein sequences using MEGA 7.0 with bootstrap = 1000 repetitions and the Poisson model [53]. The phylogenetic tree was beautified using EvolView (https://www.evolgenius.info/evolview/) online tools. The *OPR* gene subfamily of *I. batatas* was categorized based on the *A. thaliana OPR* gene subfamily.

### Collinearity analysis and Ka/Ks calculation

One Step MCSanX-Super Fast in TBtools was used to examine the duplication types and intraspecific covariance of *OPR* family members. The collinearity relationship of the *OPR* genes in sweetpotato was plotted using the advanced Circle function in TBtools. The Ka/Ks Calculator of TBtools software was used to calculate the synonymous and nonsynonymous substitution rates across paralog pairs inside *IbOPRs*.

### *Cis*-acting element analyses

The 2,000 bp promoter sequences of the sweetpotato *OPR* gene were submitted to the PlantCare website [54] (http://bioinformatics.psb.ugent.be/webtools/plantcare/html/) for predicting *cis*-acting regulatory elements based on the *I. batatas* genome database file. The results of PlantCare analysis were simplified and visualized using TBtools[51].

### RNA isolation and gene cloning

Total RNA was extracted using the Plant RNA Kit (Omega Biotech, USA), and first-strand cDNA was synthesized using TransScript® One-Step gDNA Removal and cDNA Synthesis SuperMix (TransGen Biotech, Beijing, China). Gene cloning was performed using the synthesized cDNA as a template. Specific quantitative primers (Table S7) were designed using Primer Premier 5 software based on the CDS.

A 50 μL reaction mixture was prepared, which contained 25 μL 2 × TransTaq® HiFi PCR SuperMix Master Mix, 1 μL each of the forward/reverse primers, 2 μL of cDNA template, and 21 mL of nuclease-free ddH2O. The PCR program was set as follows: predenaturation at 94 °C for 5 min, followed by 35 cycles of 94 °C for 30 s, 53 °C for 30 s, and 72 °C for 1 min, and a final extension step at 72 °C for 10 min. The PCR products were separated by 1% agarose gel electrophoresis, and the target fragment was recovered using a TIANgel Midi Purification Kit (Tiangen Biotech, Beijing, China). The target fragment was then ligated in the pEASY-T1 Cloning Kit (TransGen Biotech, Beijing, China) and transformed into competent *E. coli* DH5α. Positive monoclonal colonies detected by PCR were selected and sent to Hainan NanShan Biotech (Haikou, China) for sequencing.

### Subcellular location experiment

To confirm the location of IbOPRs, we employed the homologous recombination method to insert the full-length coding sequence (CDS) of *IbOPR2* and *IbOPR3* with a stop codon at the C-terminus of PAN580-eGFP. We employed the double digestion method to insert the CDS of *IbOPR6* and *IbOPR7* without a stop codon at the N-terminus of pCambia1300-GFP. Subsequently, we introduced the recombinant plasmid into Agrobacterium EHA105 using the freeze–thaw method. The final step involved performing subcellular localization using the tobacco protoplast method [55]. The subcellular localization experiment was conducted at Wuhan BioRun Biosciences Co. Ltd. in Wuhan, China.

### Real-time fluorescent quantitative PCR analysis (qRT‒PCR)

The Plant RNA Kit (Omega Biotech, USA) extracted total RNA from sweet potato fibrous roots treated with 200 mM NaCl in Hoagland solution for 0, 6, 12, 24, 24, 48, and 72 h. TransScript® One-Step gDNA Removal and cDNA Synthesis SuperMix (Trans Biotech, Beijing, China) was used to reverse-transcribe RNA samples. PCR was performed using particular primers on cDNA (Table S7). Each 10 μL reaction mixture contained 2.0 μL cDNA template, 5 μL 2*perfectStart Green qPCR SuperMix, 2.2 μL nucleotide-free water, and 0.4 μL 2.5 μM forward/reverse primers. MA6000 Real-Time PCR (Suzhou Molarray Co. Ltd., Suzhou, China) was used for qRT‒PCR with three biological and three technical replicates per cDNA sample. The thermal cycler was set for predenaturation at 94 °C for 30 s, 40 cycles at 5 s, 55 °C for 30 s, and 72 °C for 12 s, and dissociation at 15 s, 60 °C for 90 s, and 94 °C for 10 s. The data were quantitatively analyzed using the 2^−ΔΔCt^ method [56].

## Supplementary Information

Supplementary Material 1.

Supplementary Material 2.

Supplementary Material 3.

Supplementary Material 4.

Supplementary Material 5.

Supplementary Material 6.

Supplementary Material 7.

Supplementary Material 8.

Supplementary Material 9.

Supplementary Material 10.

Supplementary Material 11.

## Data Availability

All data generated or analysed during this study are included in this article and its supplementary information files. All materials are available through corresponding authors upon reasonable request.

## Abbreviations

OPR: 12-Oxo-phytodienoic acid reductase
*I. batatas*: *Ipomoes batatas* (L.) Lam
*A. thaliana*: *Arabidopsis thaliana*
JA: Jasmonic acid; SA: Salicylic acid
MeJA: Methyl jasmonate
ETH: Ethylene
ABA: Abscisic acid
IAA: Indole-3-acetic acid
GA: Gibberellin
GFP: Green fluorescent protein
eGFP: Enhanced green fluorescent protein
SKL: Ser-Lys-Leu
RNA: Ribonucleic acid
cDNA: Complementary DNA
qRT‒PCR: Quantitative real-time polymerase chain reaction
CDS: Coding sequence
WGD: Whole genome duplication
