# Supplementary figures and images for: Genome-wide identification, gene cloning, subcellular location and expression analysis of the OPR gene family under salt stress in sweetpotato
Source: BMC Plant Biol. 2024 Dec 6;24:1171. doi: 10.1186/s12870-024-05887-8 (PMC11622663; doi:10.1186/s12870-024-05887-8)

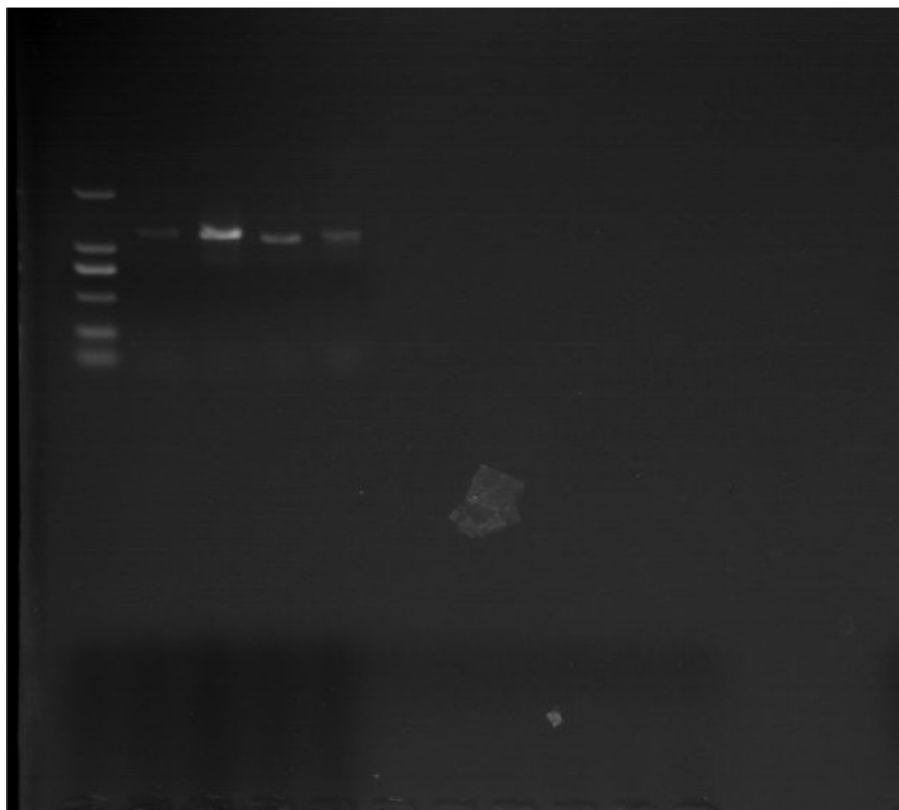

Supplement: Supplementary file 9 — Supplementary Material 9. [file 12870_2024_5887_MOESM9_ESM.pdf]

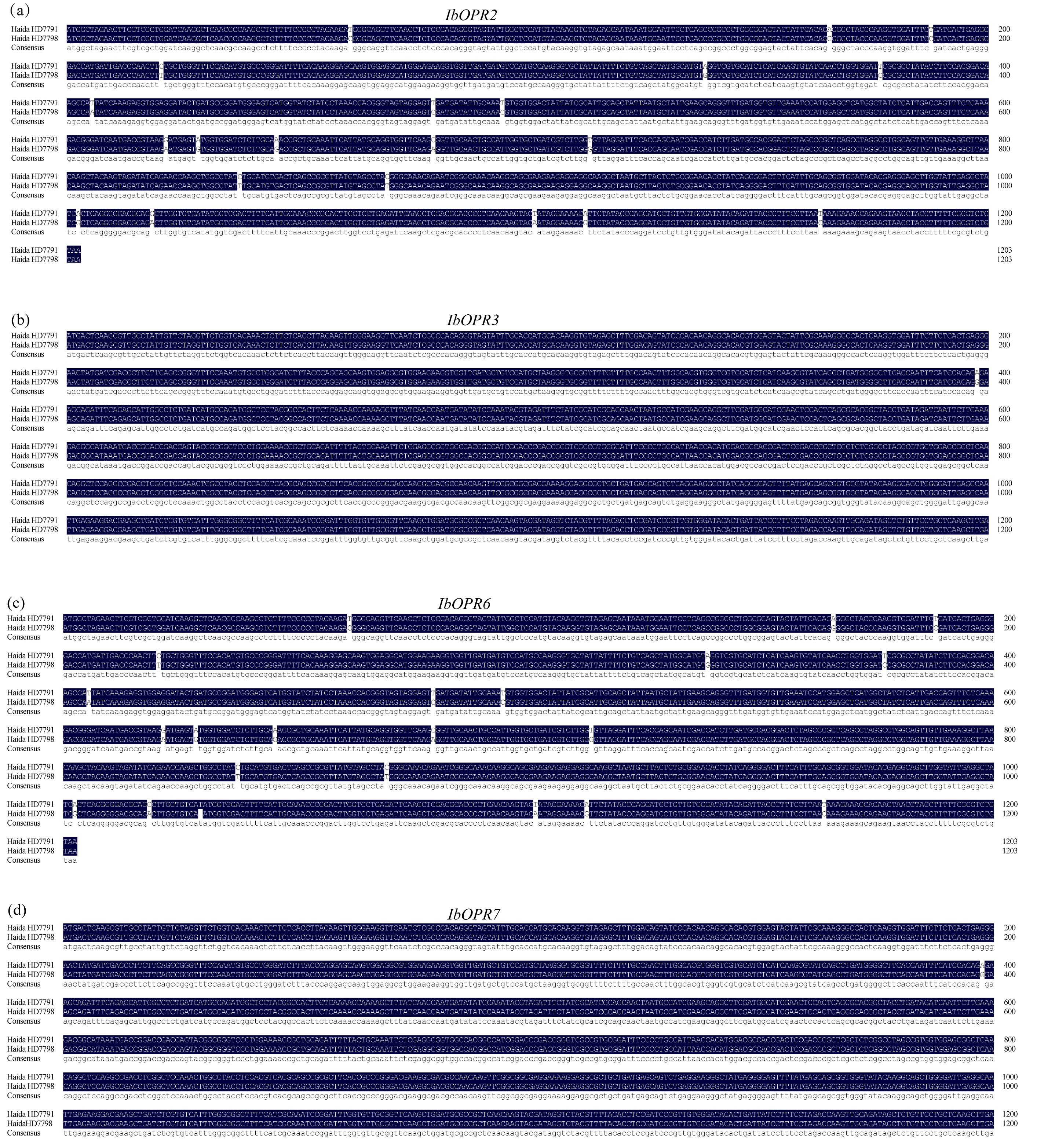

Supplement: Supplementary file 10 — Supplementary Material 10. [file 12870_2024_5887_MOESM10_ESM.jpg]

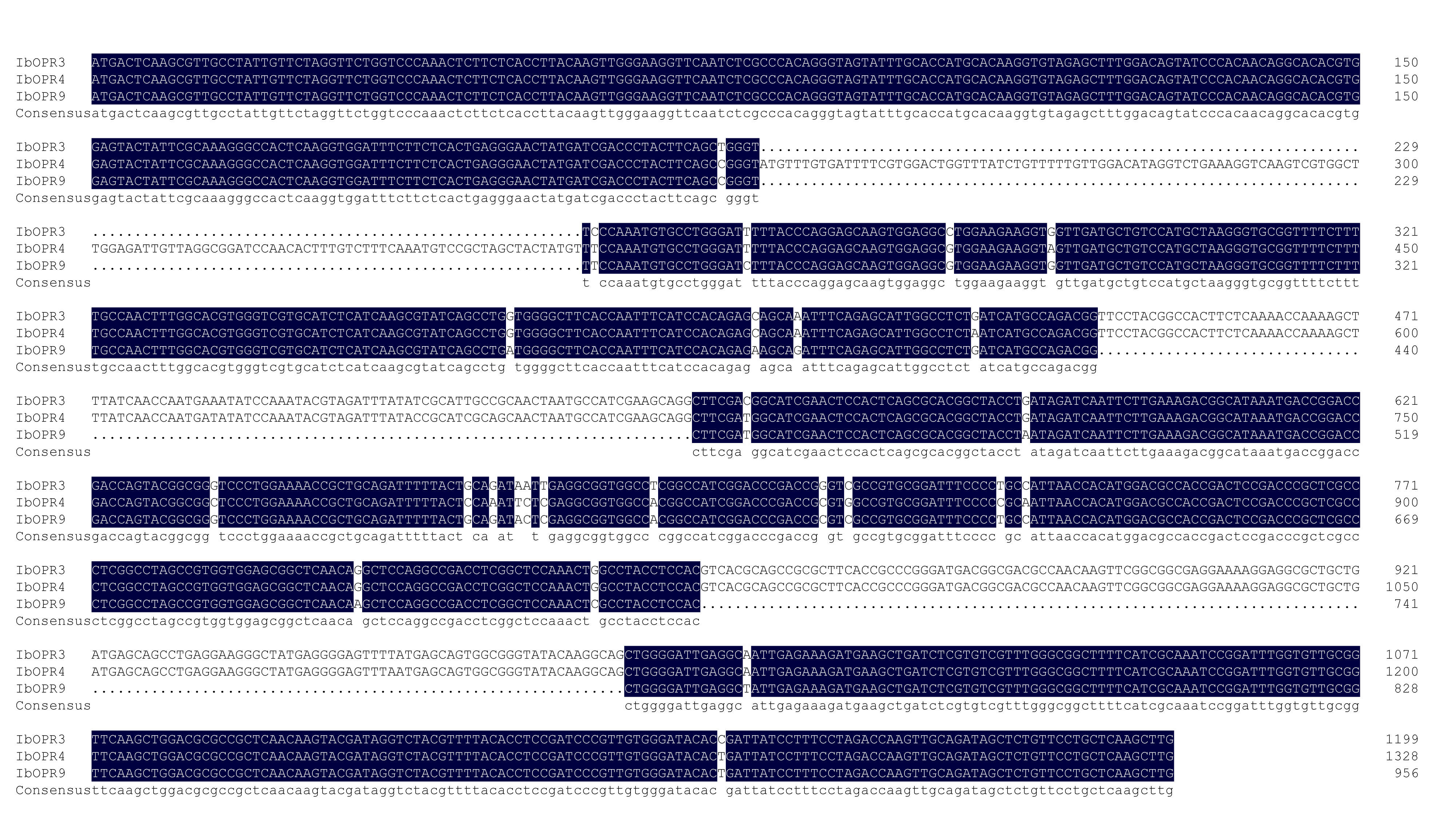

Supplement: Supplementary file 11 — Supplementary Material 11. [file 12870_2024_5887_MOESM11_ESM.tif]
